# CalFireSegNet: a lightweight hybrid attention–transformer network for post-wildfire building footprint change analysis using high-resolution satellite imagery

**DOI:** 10.1038/s41598-026-67452-7

**Published:** 2026-08-17

**Authors:** Abdullah Şener, Vedat Tümen, Burhan Ergen, Erdal Akin

**Affiliations:** 1 Management Information Systems, Faculty of Economics and Administrative Sciences, Fırat University, 23 100 Elazığ, Turkey; 2 Department of Computer Engineering, Bitlis Eren University, 13 100 Bitlis, Turkey; 3 Computer Engineering, Faculty of Engineering, Fırat University, 23 100 Elazığ, Turkey; 4 Department of Computer Science and Media Technology, Malmö University, 205 06 Malmö, Sweden

**Keywords:** Natural disasters, Disaster management, Image semantic segmentation, CalFireSegNet, Environmental sciences, Natural hazards

## Abstract

Natural disasters, particularly wildfires, cause severe human, environmental, and economic losses worldwide. Rapid and accurate identification of building footprints and potential potential structural changes is essential for effective emergency response, search-and-rescue operations, and post-disaster recovery planning. To address the challenges of identifying building loss from remote sensing imagery, this study proposes CalFireSegNet, a lightweight hybrid attention–transformer network for post-wildfire building footprint extraction and loss proxy detection from satellite imagery. The proposed architecture integrates depthwise convolutions, convolutional block attention modules (CBAM), atrous spatial pyramid pooling (ASPP), and Transformer blocks to effectively capture both local structural details and long-range contextual dependencies while maintaining low computational complexity. The model was trained and evaluated using benchmark building segmentation datasets (Inria and WHU) and subsequently applied to pre- and post-event satellite imagery from the recent California wildfires. Experimental results demonstrate that CalFireSegNet achieves superior performance compared with several state-of-the-art semantic segmentation models, including U-Net, PSPNet, DeepLabv3+, ENet, HRNet, and SegNet, obtaining 98.45% accuracy, 94.35% mIoU, and 95.03% Dice Similarity Score while requiring only 3.72 million parameters. Furthermore, a lightweight mask-difference framework was developed to generate a spatial proxy of potential building footprint loss using pre- and post-event satellite pairs. Since publicly available building-level damage annotations for recent California wildfire events remain limited, the real-world wildfire experiments are presented as a validation of cross-domain applicability rather than a fully supervised structural loss proxy estimation benchmark.

## Introduction

Natural disasters are among the most destructive events affecting human societies, causing substantial human, environmental, and economic losses worldwide every year^1,2^. In recent decades, wildfires have emerged as one of the most critical natural hazards due to their increasing frequency, intensity, and spatial extent^3^. Climate change, prolonged drought periods, rising temperatures, and extreme weather conditions have significantly increased wildfire susceptibility across many regions of the world^4^. Unlike traditional wildfire events that primarily affect forested landscapes, recent large-scale wildfires increasingly threaten urban and peri-urban environments, resulting in severe damage to residential buildings, critical infrastructure, and public facilities. Consequently, rapid and accurate post-wildfire building footprint extraction and change analysis have become essential components of disaster management, emergency response, and recovery planning^5,6^.

Accurate identification of potentially affected structures following a wildfire directly influences emergency response activities, search-and-rescue operations, resource allocation, and reconstruction planning. Conventional damage assessment approaches are generally based on field inspections and manual interpretation of aerial or satellite imagery. Although these methods can provide reliable information, they are often labor-intensive, time-consuming, costly, and difficult to implement over large geographic areas immediately after a disaster. Such limitations highlight the need for automated and scalable approaches capable of providing timely and objective building footprint extraction^7^. Recent advances in artificial intelligence (AI), particularly deep learning (DL), have significantly improved the ability to analyze remote sensing imagery for disaster-related applications. High-resolution satellite imagery, unmanned aerial vehicle (UAV) data, and other remote sensing products provide valuable information for assessing the spatial distribution and severity of disaster-induced damage. Among various computer vision techniques, semantic segmentation has become one of the most effective approaches for extracting detailed object-level information from remote sensing imagery. By assigning a class label to every pixel in an image, semantic segmentation enables precise delineation of building footprints and damaged regions, making it particularly suitable for rapid post-disaster spatial change analysis^8^.

In recent years, numerous deep learning–based studies have been conducted to automatically detect and assess building damage following natural disasters. Alisjahbana et al.^9^ integrated building detection and damage classification into a two-stage CNN approach within the xView2 Challenge and exceeded the benchmark score of 0.28 with an F1 score of 0.66. However, the authors highlighted major challenges arising from the visual similarities between damage levels and the varying distribution of damage across different disaster types. Zou et al.^10^ proposed an improved instance segmentation model incorporating the MLCA attention mechanism to rapidly and accurately determine building damage levels from high-resolution UAV imagery. Experiments conducted on data from the Yangbi earthquake demonstrated that the model achieved superior performance in both accuracy and efficiency. Zhao et al.^11^ integrated building semantic segmentation and damage classification within the ECADS-CNN architecture, which employs Efficient Channel Attention and Depthwise Separable Convolutions. The model yielded a 7.4% improvement in speed and a 5.2% increase in F1 score. Hacıefendioğlu et al.^12^ compared the FPN, U-Net, SegNet, and DeepLabV3 + architectures for detecting collapsed structures after earthquakes in Türkiye. FPN performed best in terms of accuracy and specificity, while U-Net achieved higher recall and F1 scores. Ahmadi et al.^13^ introduced a dual-branch, two-stage U-Net with a selective kernel module to reduce building footprint errors and improve damage classification. The model obtained high F1 and IoU values on the xBD dataset, and its transferability was validated using the 2003 Bam earthquake. Ro and Gong^14^ presented a novel workflow that combines large-scale pretrained instance segmentation models and transformer-based detection networks for detailed post-hurricane damage analysis. Their approach enabled component-level segmentation—such as roofs, windows, and walls—and established a comprehensive digital damage assessment framework. Wiguna et al.^15^ evaluated the performance of transformer-based models on the 2011 Tohoku Tsunami and the 2023 Türkiye–Syria Earthquake. While high accuracy was achieved using cross-entropy and focal loss, a notable performance drop was observed in the moderate damage class. Neto and Dantas^16^ enhanced U-Net with a BDANet backbone and morphological operations, developing a high-accuracy model for post-disaster damage assessment that outperformed all compared methods. Ahn et al.^17^ introduced DAVI, an approach that operates without requiring local labels. Based on a Vision Foundation Model, the method transfers knowledge from the source to the target region through a two-stage adaptation process and produced successful results. Lu et al.^18^ proposed a transformer-based model incorporating a bitemporal attention module and sequential regression loss to improve the tasks of building change detection (BCD) and building damage assessment (BDA). The model achieved state-of-the-art accuracy across three major datasets. Chicchon et al.^19^ developed an EfficientNet-based U-Net for building footprint segmentation and change detection in cultural heritage sites, achieving approximately 70% IoU on the SpaceNet dataset. This work provides an important example for the long-term monitoring of post-disaster changes. Wang et al.^20^ integrated building extraction and damage assessment within PCDASNet, a Siamese network incorporating a position-constrained differential attention mechanism. The model achieved an F1 score exceeding 73%, outperforming existing approaches. Qiao et al.^21^ proposed a weakly supervised structural loss proxy estimation method using pre- and post-disaster high-resolution imagery. Experiments on the 2010 Haiti earthquake dataset demonstrated higher accuracy compared to existing models. Zheng et al.^22^ introduced FireDM, a diffusion-based data generation method designed to address data scarcity in fire scene segmentation. The synthetic wildfire images and masks produced by the framework outperformed real datasets in accuracy, IoU, and F1 metrics, offering an important contribution to overcoming data limitations for post-wildfire structural loss proxy estimation. A summary of these studies is presented in Table 1.

**Table 1:** A comprehensive review of studies on the detection of building damage through remote sensing imagery.

Authors	Year	Dataset	Decision method
Alisjahbana et al.^9^	2024	xBD dataset	DeepDamageNet
Zou et al.^10^	2024	Yangbi dataset	MLCA (Mixed Local Channel Attention)
Zhao et al.^11^	2024	xBD dataset	ECADS-CNN
Hacıefendioğlu et al.^12^	2024	Southeast Turkey Earthquake Region Satellite Images	U-Net, LinkNet, FPN, PSPNet
Ahmadi et al.^13^	2024	xBD dataset	BD-SKUNet
Ro and Gong^14^	2024	Hurricane Harvey Dataset	Transformer-based fine-tuning object detection
Wiguna et al.^15^	2024	2011 Tohoku Tsunami, 2023 Turkey-Syria Earthquake Dataset	Transformer-based model
Neto and Dantas^16^	2024	xBD Dataset	BDANet
Ahn et al.^17^	2024	xBD Dataset	DAVI (Disaster Assessment with Vision Foundation Model)
Lu et al.^18^	2024	LEVIR-CD+, S2Looking ve xBD Dataset	Bitemporal Attention Transformer (BAT)
Chicchon et al.^19^	2024	SpaceNet V1 (Rio), SpaceNet V2 (Shanghai)	UNet (EfficientNet B0-B7 encoder)
Wang et al.^20^	2024	xBD dataset and xFBD dataset	PCDASNet
Qiao et al.^21^	2024	Haiti 2010 Remote Sensing Images	Weakly Supervised Method, FCN (Fully Convolutional Network)
Zheng et al.^22^	2024	FireDM-generated Fire Segmentation Dataset	FireDM, Stable Diffusion, ChatGPT4-Fire

As summarized in Table 1, existing studies have achieved remarkable success in building damage assessment using deep learning techniques. However, most approaches either rely on computationally intensive architectures or focus primarily on improving accuracy without considering deployment efficiency. Furthermore, limited attention has been paid to developing lightweight frameworks capable of simultaneously capturing local structural details and global contextual information for rapid building footprint extraction and loss proxy detection in post-wildfire environments. These limitations motivate the development of a more efficient and robust segmentation framework for wildfire-related building footprint analysis.

Despite the significant progress achieved by existing approaches, several important challenges remain unresolved. First, many state-of-the-art architectures achieve high segmentation accuracy at the cost of increased computational complexity and large numbers of trainable parameters, limiting their deployment in time-critical disaster scenarios. Second, while attention mechanisms and transformer-based models have demonstrated strong capabilities in capturing contextual information, their integration into lightweight building segmentation frameworks remains relatively limited. Third, post-wildfire building extraction requires the simultaneous extraction of fine-grained structural details and long-range contextual dependencies, which conventional convolutional architectures often struggle to model effectively.

Recent studies have demonstrated the effectiveness of hybrid CNN–Transformer architectures combined with attention mechanisms and multi-scale feature extraction strategies. For example, Li et al.^23^ proposed RCCT-ASPPNet, a dual-encoder remote sensing segmentation framework that integrates Transformer-based feature fusion, ASPP, and CBAM to improve semantic segmentation performance. Although this approach achieved promising results on remote sensing datasets, its dual-encoder design introduces additional architectural complexity and computational overhead. Similarly, Song et al.^24^ introduced TransUNet-CBAM, a hybrid Transformer and CBAM-based segmentation framework for cardiovascular structure segmentation in medical images. Their findings demonstrated the benefit of combining global contextual modeling with attention-based feature refinement. However, this architecture was specifically optimized for medical image characteristics and does not address the computational constraints and domain-specific challenges encountered in post-disaster remote sensing applications.

In contrast to these approaches, CalFireSegNet does not aim to introduce individual novel components, since Depthwise Convolution (DWConv), CBAM, ASPP, and Transformer mechanisms have already been extensively investigated. Instead, the main contribution of this study lies in the design of a lightweight and application-oriented hybrid architecture that efficiently integrates these components for post-wildfire building footprint extraction. By combining parameter-efficient convolutional operations with localized Transformer-based contextual modeling, CalFireSegNet achieves a favorable balance between segmentation accuracy and computational efficiency, enabling its potential deployment in rapid disaster assessment scenarios.

To address these challenges, this study proposes CalFireSegNet, a lightweight hybrid attention–transformer for post-wildfire building footprint extraction and loss proxy detection from satellite imagery. The proposed architecture combines DWConv, Convolutional Block Attention Modules (CBAM), Atrous Spatial Pyramid Pooling (ASPP), and Transformer blocks within an encoder–decoder framework. This design enables efficient extraction of local structural features, multi-scale contextual representations, and global spatial dependencies while maintaining low computational complexity. The main contributions of this study are summarized as follows:

- A novel lightweight semantic segmentation architecture, termed CalFireSegNet, is proposed for high-precision building footprint extraction and post-wildfire change analysis from satellite imagery.
- A hybrid feature extraction strategy integrating DWConv and CBAM is developed to improve feature representation capability while maintaining a low-parameter architecture.
- ASPP and Transformer modules are jointly incorporated to capture both multi-scale contextual information and long-range spatial dependencies in complex disaster environments.
- A lightweight building loss proxy pipeline based on pre- and post-event satellite imagery is introduced to estimate potential structural footprint changes through mask-based set-difference analysis. This pipeline is designed as an initial rapid assessment tool rather than a supervised building damage severity classification framework.
- Experimental evaluations indicate that CalFireSegNet achieves competitive or superior performance compared with several widely used semantic segmentation architectures, including U-Net, PSPNet, DeepLabv3+, ENet, HRNet, and SegNet, while preserving computational efficiency and lightweight characteristics.

The proposed framework provides an effective and practical solution for rapid building footprint extraction and initial loss proxy estimation, serving to support emergency response operations, resource allocation strategies, and initial disaster management decision-making processes.

The remainder of this paper is organized as follows. Section  2 presents the datasets, preprocessing procedures, and the details of the proposed CalFireSegNet architecture, including the integrated CBAM, Depthwise Convolution, ASPP, and Transformer modules. Section  3 describes the experimental setup, training strategy, evaluation metrics, quantitative comparisons with benchmark segmentation models, and the cross-domain wildfire application analysis using California wildfire satellite imagery. Section  4 discusses the strengths, limitations, and practical considerations of the proposed framework, while Sect.  5 concludes the study by summarizing the main findings and outlining future research directions.

## Materials and methods

This section presents the characteristics of the imagery datasets employed in the study, the applied preprocessing pipeline, and the architectural details of the proposed lightweight hybrid network, CalFireSegNet, designed for rapid post-wildfire building change analysis.

### Datasets

In this study, two benchmark datasets were employed to train and evaluate the semantic segmentation performance of CalFireSegNet for building footprint extraction, while an additional high-resolution satellite imagery dataset was utilized to validate its effectiveness in real-world post-wildfire building change analysis.

The first benchmark dataset employed in this study is the Inria Aerial Image Labeling Dataset^25^, which consists of 360 high-resolution orthorectified RGB tiles covering a total area of 810 km². The dataset is equally divided into training/validation and testing subsets, each representing an area of 405 km². The imagery encompasses ten diverse urban and peri-urban regions worldwide, with a ground sampling distance (GSD) of 30 cm per pixel. To ensure compatibility with the computational requirements of deep learning architectures, the original 5000 × 5000 pixel tiles were partitioned into non-overlapping 1024 × 1024 pixel patches and subsequently resized to 256 × 256 pixel inputs. The dataset can be accessed via the following link: https://www.kaggle.com/datasets/huanranye/inria-aerial-image-labeling-dataset?select=cropped_1024.

The second benchmark dataset utilized in this study is the WHU Building Dataset^26^, which includes the aerial imagery subset acquired over Christchurch, New Zealand. This dataset contains more than 220,000 individual building instances distributed across an area of approximately 450 km², with an ultra-high spatial resolution of 0.075 m. Additionally, the dataset provides a satellite imagery subset covering regions in East Asia with a ground sampling distance (GSD) of 2.7 m. To maintain a consistent input configuration for the proposed model, the original RGB image patches of 512 × 512 pixels were resized using bilinear interpolation to 256 × 256 × 3 inputs, corresponding to the standard input tensor size ($x\in{R}^{256x256x3}$) of CalFireSegNet.The dataset can be accessed through the following link: https://www.kaggle.com/datasets/sengulgs/whu-building-dataset.

Experimental Protocol. To ensure a fair comparison, all segmentation models were trained and evaluated using exactly the same experimental protocol. The benchmark datasets were partitioned once into fixed training, validation, and testing subsets, and these subsets were consistently used throughout all experiments without modification. Model selection was performed according to the validation performance during training, whereas all quantitative performance metrics reported in this study were computed exclusively on the independent test set. Furthermore, identical preprocessing procedures, input image dimensions, optimizer settings, learning-rate schedule, and evaluation metrics were adopted for every compared model to guarantee a consistent and unbiased experimental evaluation.

**Fig. 1:**
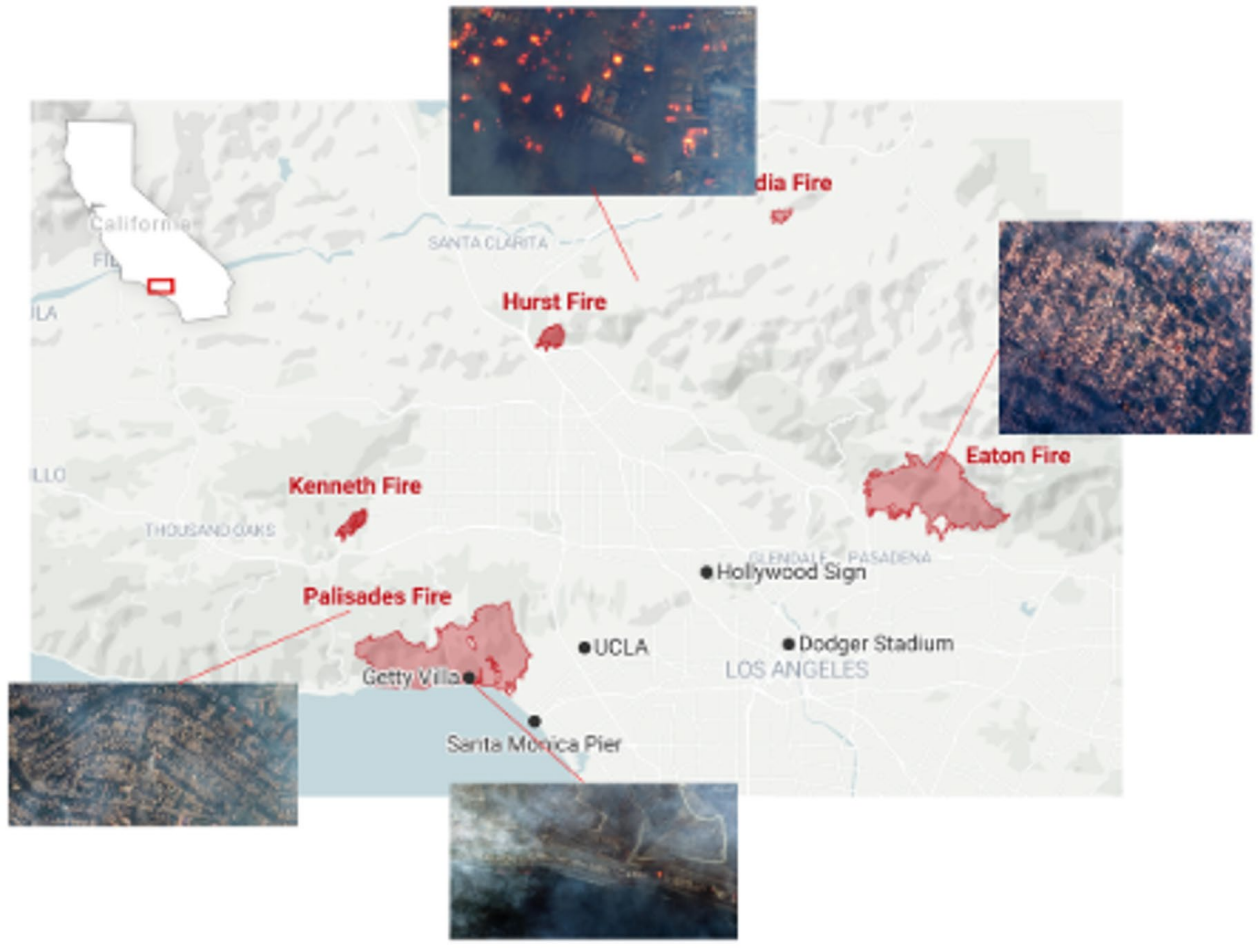
Representative pre- and post-wildfire satellite images of fire-affected areas in Los Angeles, California. The images were obtained from the Maxar Open Data Program and used in this study for cross-domain evaluation.

For real-world applicability analysis under post-disaster conditions, multi-temporal high-resolution satellite imagery associated with severe wildfire events in California was collected from the Maxar Open Data Program^27^. The dataset consists of four pre-disaster and four post-disaster orthorectified image pairs representing affected urban–forest interface regions in Los Angeles, CA. To reduce spatial inconsistencies caused by off-nadir satellite acquisition angles, the pre- and post-event images were co-registered using mutual information-based cross-correlation. Subsequently, the aligned image pairs were divided into 256 × 256 pixel patches and used during the inference phase of the trained CalFireSegNet model to investigate the transferability of the learned building representation to an unseen wildfire domain. Representative examples of wildfire imagery from different regions of Los Angeles are presented in Fig. 1.

### Convolutional block attention module (CBAM)

The convolutional block attention module (CBAM) is a mechanism that applies attention over feature maps within deep learning architectures, enabling the model to learn discriminative information more effectively^28^. This module enhances the prominence of significant features during the learning process while suppressing the influence of relatively less important information. The CBAM architecture consists of two sequential components: Channel Attention and Spatial Attention. The Channel Attention module aims to determine the relative importance of each channel within the input feature map. In this stage, global average pooling and global max pooling operations are applied channel-wise to the input feature map $F\in{R}^{HXWXC}$. These operations are expressed as shown in Eq. 1.

1 $${F}_{avg}^{c}=\frac{1}{H.W}\sum_{i=1}^{H}\sum_{j=1}^{W}F\left(i,j,c\right),{F}_{max}^{c}=\genfrac{}{}{0pt}{}{max}{i,j}F(i,j,c)$$

The summary vectors obtained through Eq. 1 are passed through a two-layer fully connected (dense) structure with shared weights to generate the channel attention mask. This process yields the channel attention mask ${M}_{c}\left(F\right)$, as presented in Eq. 2.

2 $${M}_{c}\left(F\right)=\sigma({W}_{1}\left({W}_{0}\left({F}_{avg}^{c}\right)\right)+{W}_{1}\left({W}_{0}\left({F}_{max}^{c}\right)\right))$$

In Eq. 2, the weights ${W}_{0}$ and ${W}_{1}$ represent the transformations that reduce and subsequently restore the channel dimensionality, respectively, while denotes the sigmoid activation function. The resulting channel attention mask is then multiplied element-wise with the input feature map to obtain the updated feature map ${F}^{{\prime}}$, as shown in Eq. 3.

3 $${F}^{{\prime}}={M}_{c}\left(F\right)\odot F$$

The spatial attention module focuses on identifying which spatial regions within the feature map are more informative. To achieve this, average pooling and max pooling operations are applied along the channel dimension of the feature map ${F}^{{\prime}}$, obtained from Eq. 3, resulting in two distinct spatial summary maps. This process is formulated as shown in Eq. 4.

4 $${F}_{avg}^{{\prime}}=\frac{1}{C}\sum_{k=1}^{C}{F}^{{\prime}}\left(i,j,k\right),{F}_{max}^{{\prime}}=\genfrac{}{}{0pt}{}{max}{k}F{\prime}(i,j,k)$$

The two summary maps obtained in Eq. 4 are then concatenated along the channel dimension and processed with a 7 × 7 convolutional filter to generate the spatial attention mask. Through this operation, the spatial attention mask ${M}_{s}\left({F}^{{\prime}}\right)$ is produced, as shown in Eq. 5.

5 $${M}_{s}\left({F}^{{\prime}}\right)=\sigma\left({f}^{7x7}\left(\left[{F}_{avg}^{{\prime}};{F}_{max}^{{\prime}}\right]\right)\right)$$

The spatial attention mask obtained in Eq. 5 is applied to the feature map through an element-wise multiplication, resulting in the final feature map $F{\prime}{\prime}$, as presented in Eq. 6.

6 $${F}^{{\prime}{\prime}}={M}_{s}\left(F{\prime}\right)\odot F{\prime}$$

**Fig. 2:**
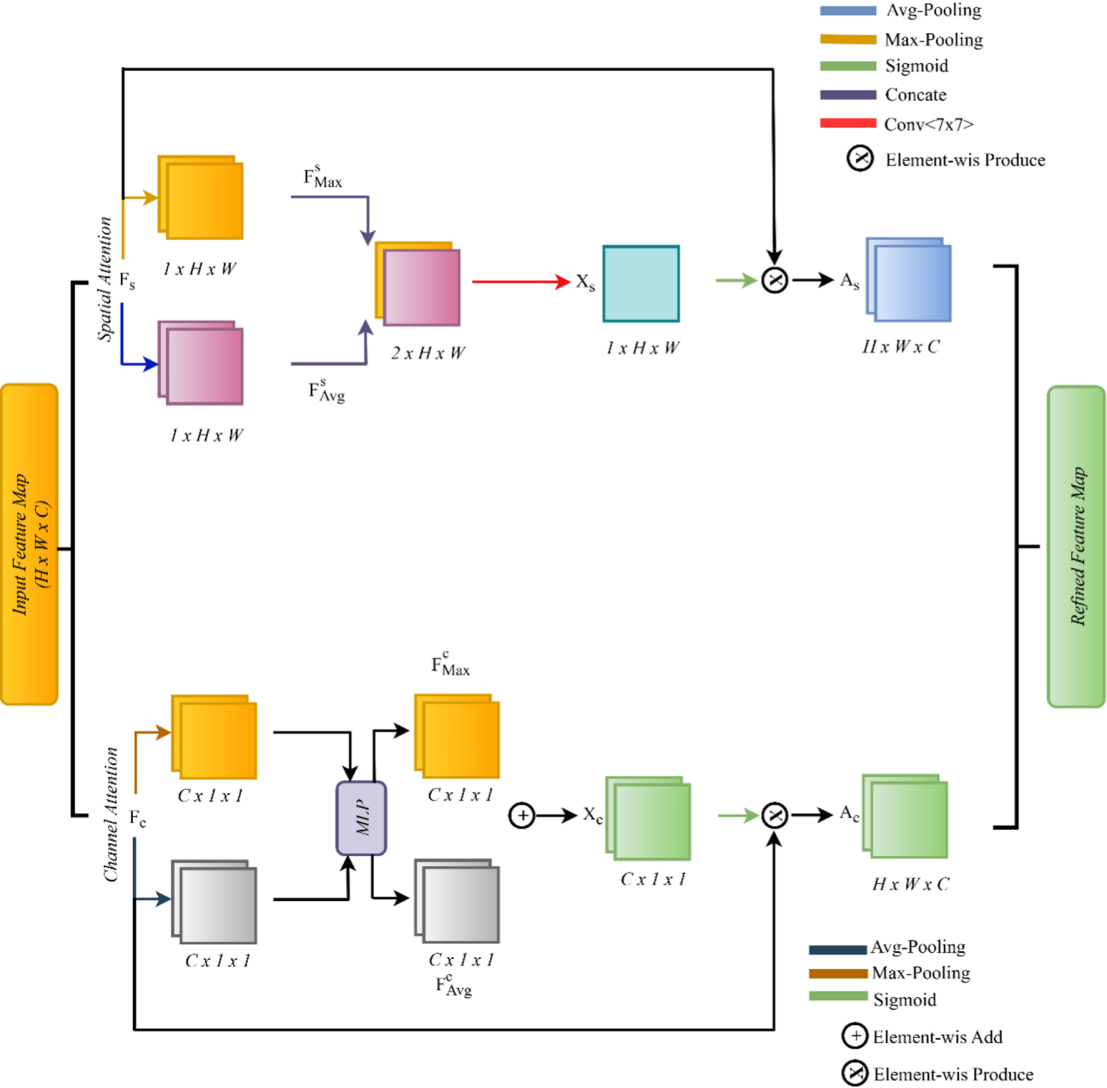
Convolutional block attention module (CBAM).

The overall architecture of the convolutional block attention module (CBAM) used in this study is schematically illustrated in Fig. 2.

### Depthwise convolution block

The Depthwise Convolution Block is a lightweight and efficient convolutional approach that enables feature extraction at a significantly lower computational cost compared to traditional convolution operations. This structure aims to reduce the number of parameters and computational burden by decomposing the convolution process into two stages: DWConv and pointwise convolution. In the DWConv stage, each channel is processed independently from the others^29^. For an input feature map $F\in{R}^{HxWxC}$, a separate *KxK* filter is applied to each channel, and this operation is expressed by Eq. 7.

7 $${F}_{k}^{\left(d\right)}={F}_{k}*{K}_{k},k=\mathrm{1,2},\dots\dots.,C$$

In Eq. 7, the operator * denotes the convolution operation, while ${K}_{k}$ represents the learned filter kernel for the *k*- th channel. As a result of this stage, the number of channels is preserved, and each channel is processed independently with its corresponding filter kernel. In the second stage, known as the pointwise convolution step, 1 × 1 filters are used to integrate information across channels and to transform the number of output channels to the desired dimension. This operation is presented in Eq. 8.

8 $${F}^{\left(p\right)}=\sum_{k=1}^{C}{F}_{k}^{\left(d\right)}*{P}_{k}$$

**Fig. 3:**
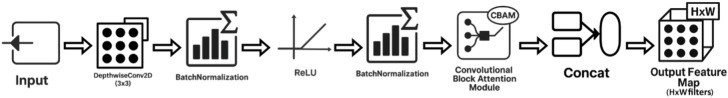
Integrated architecture of the depthwise convolution block with the convolutional block attention module (CBAM).

In Eq. 8, ${P}_{k}$ denotes the 1 × 1 convolution filter for the *k*-th channel. Through this stage, information extracted from different channels is integrated to construct a richer and more expressive output feature map. The structure of the DWConv Block integrated with the Convolutional Block Attention Module (CBAM), as utilized in this study, is presented in Fig. 3.

### Atrous spatial pyramid pooling (ASPP)

Atrous Spatial Pyramid Pooling (ASPP)^30^ is a feature extraction technique that employs parallel atrous (dilated) convolution layers with different dilation rates to capture multi-scale contextual information. It is particularly effective in semantic segmentation problems where both local and global context must be jointly utilized. As one of the core components of the DeepLab architectures, ASPP enables the integration of feature maps at multiple resolutions, allowing the model to more accurately detect objects of varying scales. In standard convolution, the kernel interacts with adjacent pixels on the input feature map. In contrast, atrous convolution expands the receptive field by inserting gaps between kernel elements. This allows the model to capture a broader context without increasing the number of parameters or computational cost. Mathematically, an atrous convolution operation is expressed as shown in Eq. 9.

9 $$y\left[i\right]=\sum_{k=1}^{K}x\left[i+r.k\right].w\left[k\right]$$

In Eq. 9, *x* denotes the input feature map, $w\left[k\right]$ represents the *k-* -th kernel weight, r refers to the dilation rate, *K* is the kernel size, and $y\left[i\right]$ indicates the output feature value. When *r=1*, the operation reduces to standard convolution, whereas for *r>1*, *r-1* empty spaces are inserted between kernel elements.

The ASPP module applies multiple atrous convolution layers with different dilation rates in parallel, thereby extracting contextual information at various scales. Within the ASPP structure, the 1 × 1 convolution preserves local contextual information, while the 3 × 3 atrous convolutions (with different r values) capture medium- and large-scale context. The Global Average Pooling (GAP) operation incorporates global contextual information extracted from the entire image. Finally, concatenation followed by a 1 × 1 convolution integrates the multi-scale features and performs dimensionality reduction. Mathematically, the ASPP module can be modeled over the input feature map F as shown in Eq. 10.

10 $$ASPP\left(F\right)={\upphi}(\sum_{m=1}^{M}{Conv}_{{k}_{m},{r}_{m}}\left(F\right)\oplus GAP\left(F\right)$$

**Fig. 4:**
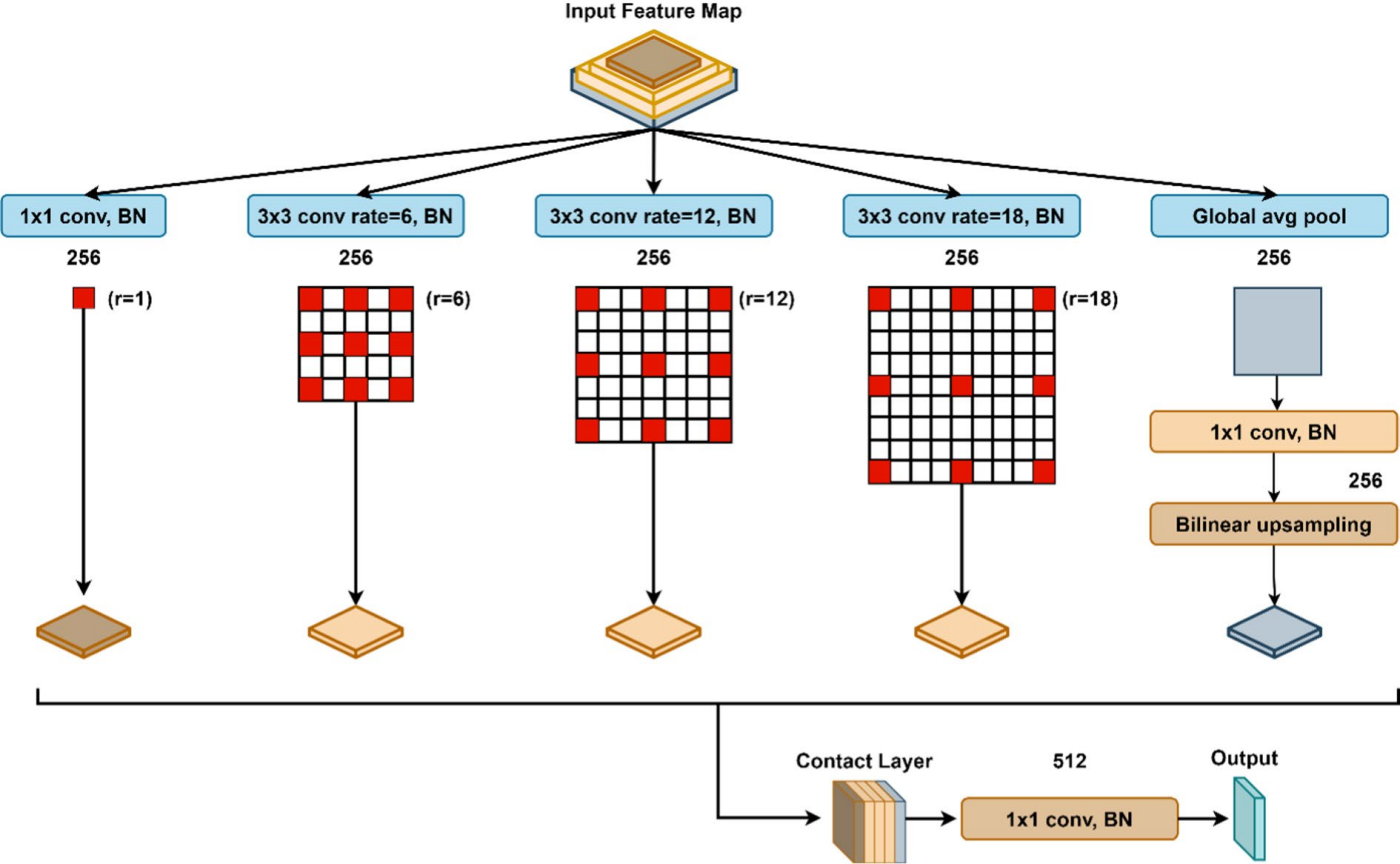
Atrous spatial pyramid pooling^28^.

In Eq. 10, ${Conv}_{{k}_{m},{r}_{m}}$ denotes an atrous convolution with a kernel size of ${k}_{m}x{k}_{m}$ and dilation rate ${r}_{m}$; $GAP\left(F\right)$ represents the global contextual information obtained via global average pooling; ⊕ indicates channel-wise concatenation; ϕ(⋅) corresponds to the 1 × 1 convolution and activation function applied to the concatenated features; and *M* enotes the number of parallel atrous convolution branches. The general structure of the Atrous Spatial Pyramid Pooling module is illustrated in Fig. 4.

### Transformer block

The Transformer block is employed to model spatial relationships within the feature maps extracted from the image. It takes as input a feature map $X\in{R}^{HxWxC}$ and produces an output of the same dimensions, $\stackrel{\sim}{X}\in{R}^{HxWxC}$. As shown in Eq. 11, the block first converts the 2D feature map into a sequence of vectors.

11 $${X}_{seq}=Reshape\left(X\right)\in{R}^{\left(H.W\right)xC}$$

In Eq. 11, *H* and *W* denote the height and width dimensions, respectively, while *C* represents the number of channels. As shown in Eq. 12, normalization is applied within the Transformer block to stabilize the learning process.

12 $$\widehat{X}=LayerNorm\left({X}_{seq}\right)$$

The attention mechanism learns the relationship of each position with all other positions. Multi-Head Attention is formulated as shown in Eq. 13.

13 $$Attention\left(Q,K,V\right)=Softmax\left(\frac{Q{K}^{T}}{\sqrt{{d}_{k}}}\right)V$$

$$MHA\left(\widehat{X}\right)=Concat\left({head}_{1},\dots,{head}_{h}\right){W}_{0}$$.

In Eq. 13, *Q,K* and *V* represent the query, key, and value matrices, respectively; ${d}_{k}$ denotes the dimensionality of the key vectors, and ℎ represents the number of heads. The output is then added to the input through a residual connection, as shown in Eq. 14.

14 $${X}_{1}={X}_{seq}+MHA\left(\widehat{X}\right)$$

In the next stage, a two-layer feed-forward network is applied to the attention outputs, as shown in Eq. 15.

15 $$FFN\left({X}_{1}\right)={W}_{2}\sigma\left({W}_{1}{X}_{1}+{b}_{1}\right)+{b}_{2}$$

In Eq. 15, *σ* denotes the *GELU* activation function. The output is then combined again with a residual connection, as illustrated in Eq. 16.

16 $${X}_{out}={X}_{1}+FFN\left({X}_{1}\right)$$

Finally, the sequence structure is converted back into a 2D feature map, as shown in Eq. 17.

17 $$\stackrel{\sim}{X}=Reshape\left({X}_{out}\right)\in{R}^{HxWxC}$$

**Fig. 5:**
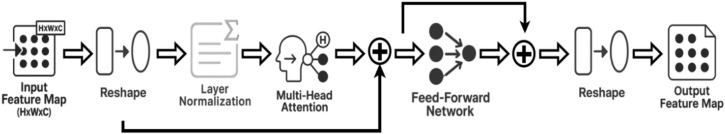
The overall architecture of the transformer block.

The overall architecture of the transformer block is illustrated in Fig. 5.

### Proposed segmentation model (CalFireSegNet)

In this study, a novel deep learning–based segmentation model named CalFireSegNet is proposed to accurately extract post-wildfire building footprints. The model adopts an encoder–decoder architecture composed of innovative structural components that integrate attention mechanisms and multi-scale contextual information. In the encoder section, four sequential DWConv blocks are employed. Each block is enhanced with a Convolutional Block Attention Module (CBAM), which incorporates both channel and spatial attention mechanisms. The CBAM structure assigns importance weights at the channel and spatial levels on the input feature maps, thereby highlighting discriminative structural features while suppressing irrelevant information. Through max-pooling operations applied in the encoder layers, feature maps are progressively downsampled, enabling the model to learn broader contextual information. During this process, intermediate feature maps are preserved via skip connections and later reused in the decoder to maintain fine-grained spatial details. The features extracted at the encoder output are forwarded to the Atrous Spatial Pyramid Pooling (ASPP) block to effectively capture multi-scale contextual information. By utilizing convolutions with different dilation rates, the ASPP block is capable of simultaneously detecting both small-scale structures and large, spatially distributed building complexes, combining this multi-scale information into a unified representation. The multi-scale features produced by ASPP are then processed by a Transformer block to model long-range dependencies and learn global contextual relationships. Through its Multi-Head Attention mechanism, the Transformer captures interactions among built-up regions spread across wide areas, thereby contributing to improved segmentation performance. In the decoder section, the skip connections stored during the encoder stage are fused with depthwise-CBAM blocks to progressively upsample the feature maps. As spatial resolution is increased through up-sampling, residual connections and attention mechanisms help preserve even small and scattered building patterns and make them more distinguishable. Finally, the segmentation output is generated through a 1 × 1 convolution followed by a sigmoid activation function, enabling binary classification of pixels into building footprint and background (non-building).

**Fig. 6:**
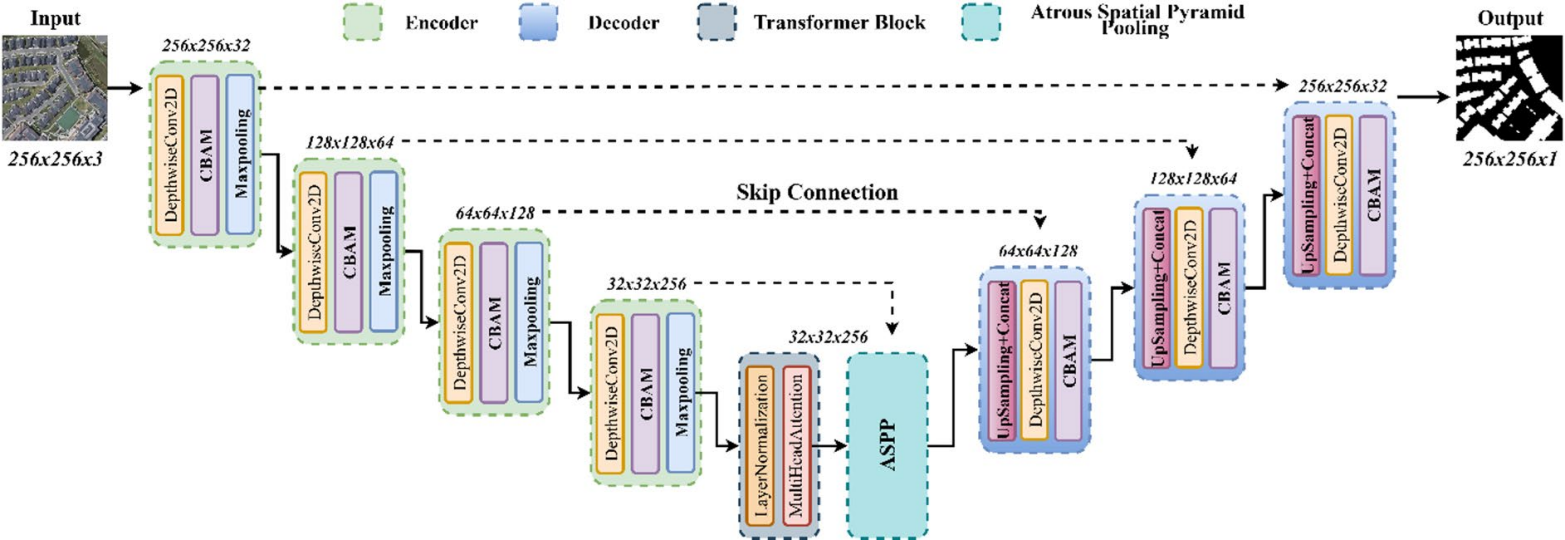
Overall architecture of the proposed CalFireSegNet model. The figure was designed and generated by the authors.

The proposed CalFireSegNet model offers high accuracy and reliability in post-wildfire building footprint extraction by effectively detecting structures at multiple scales, leveraging both local and global contextual information, and preserving spatial details through residual–skip connections. The overall architecture of the model and the functioning of each structural component are presented in detail in Fig. 6.

To ensure structural efficiency and avoid architectural redundancy, CalFireSegNet is built upon a progressive module-integration strategy where each component addresses a distinct spatial modeling challenge:

- Convolutional Backbone: Functions as the primary local feature extractor, capturing fine-grained texture details and basic structural outlines of buildings from high-resolution satellite imagery.
- Hybrid Attention Module: Focuses specifically on noise suppression and boundary refinement. In post-wildfire scenarios, charred ground, ash, and shadows frequently produce false-positive activations in standard CNNs. This module dynamically recalibrates spatial and channel feature maps, ensuring high-response weights are restricted to distinct building structures and refined object boundaries.
- Lightweight Transformer Block: Overcomes the inherently limited receptive field of local convolutions by establishing long-range contextual dependencies across the visual scene. This enables the network to accurately differentiate visually ambiguous building footprints from complex surrounding terrain and fire-affected background features based on global spatial context.

Through this functional division, each module contributes a distinct and non-redundant capability, maximizing segmentation accuracy while maintaining a lightweight computational footprint.

## Experimental results

**Fig. 7:**
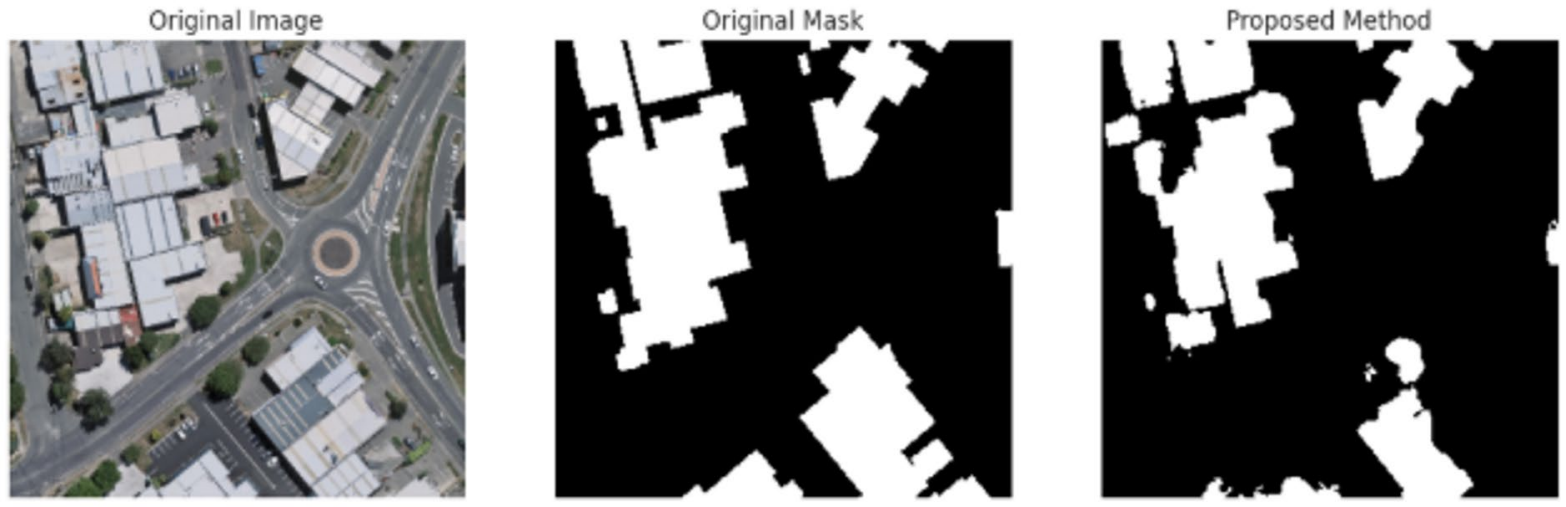
Visualization of building segmentation masks generated by the proposed CalFireSegNet model on the test dataset. The results were generated by the authors using the proposed framework.

In this study, the proposed model is designed to first learn to accurately detect buildings from satellite imagery, and subsequently perform a segmentation task to extract building footprints using pre-fire and post-fire image pairs. The proposed approach is built upon a deep learning-based semantic segmentation architecture, named CalFireSegNet, which is trained for building footprint extraction and subsequently investigated for post-wildfire structural loss proxy estimation. The model is trained and evaluated using the Inria Aerial Image Labeling Dataset and the WHU Building Dataset, both offering a high spatial resolution of 0.3 m per pixel (Ground Sampling Distance - GSD). This high spatial resolution ensures that fine-grained structural details, building boundaries, and rooftop features are preserved during feature extraction. The performance of CalFireSegNet is assessed in comparison with widely used and contemporary segmentation architectures in the literature, including U-Net, PSPNet, DeepLabv3+, ENet, HRNet, and SegNet. Example predicted mask outputs generated during the model’s testing phase are presented in Fig. 7.

The training of the CalFireSegNet model was conducted in the Google Colab computational environment using the Python programming language and the TensorFlow and Keras deep learning libraries. In the optimization phase, the Adam optimizer was selected due to its advantages in fast convergence and adaptive learning. To dynamically adjust the learning rate throughout the training process, the ReduceLROnPlateau learning rate scheduling strategy was applied. ReduceLROnPlateau is an adaptive learning rate scheduling technique that automatically reduces the learning rate when the monitored validation metric stops improving for a predefined number of epochs, allowing more stable convergence during training^31^. The initial learning rate was set to 0.01, while the minimum learning rate was defined as 0.00001. The model was trained for 100 epochs with a batch size of 16. Throughout the training process, validation data were continuously monitored to prevent overfitting and to ensure the selection of optimal model weights. The performance of CalFireSegNet in post-fire structural loss proxy estimation was quantitatively evaluated using Accuracy, Precision, Recall, F1-Score, Dice Similarity Score (DSS), and Mean Intersection over Union (mIoU) metrics. These evaluation measures were computed based on true positive (TP), false positive (FP), true negative (TN), and false negative (FN) values, and their mathematical definitions are presented in Eqs. (18–23).

18 $$Acc=\frac{TP+TN}{TP+TN+FP+FN}$$

19 *Prec=TP/(TP+FP)*

20 *Rec=TP/(TP+FN)*

21 $$F1-Scr=\frac{2xTP}{2xTP+FP+FN}$$

22 $$DiceSS=\frac{2.PrecxRec}{Prec+Rec}$$

23 $$mIoU=\frac{TP}{TP+FP+FN}$$

**Fig. 8:**
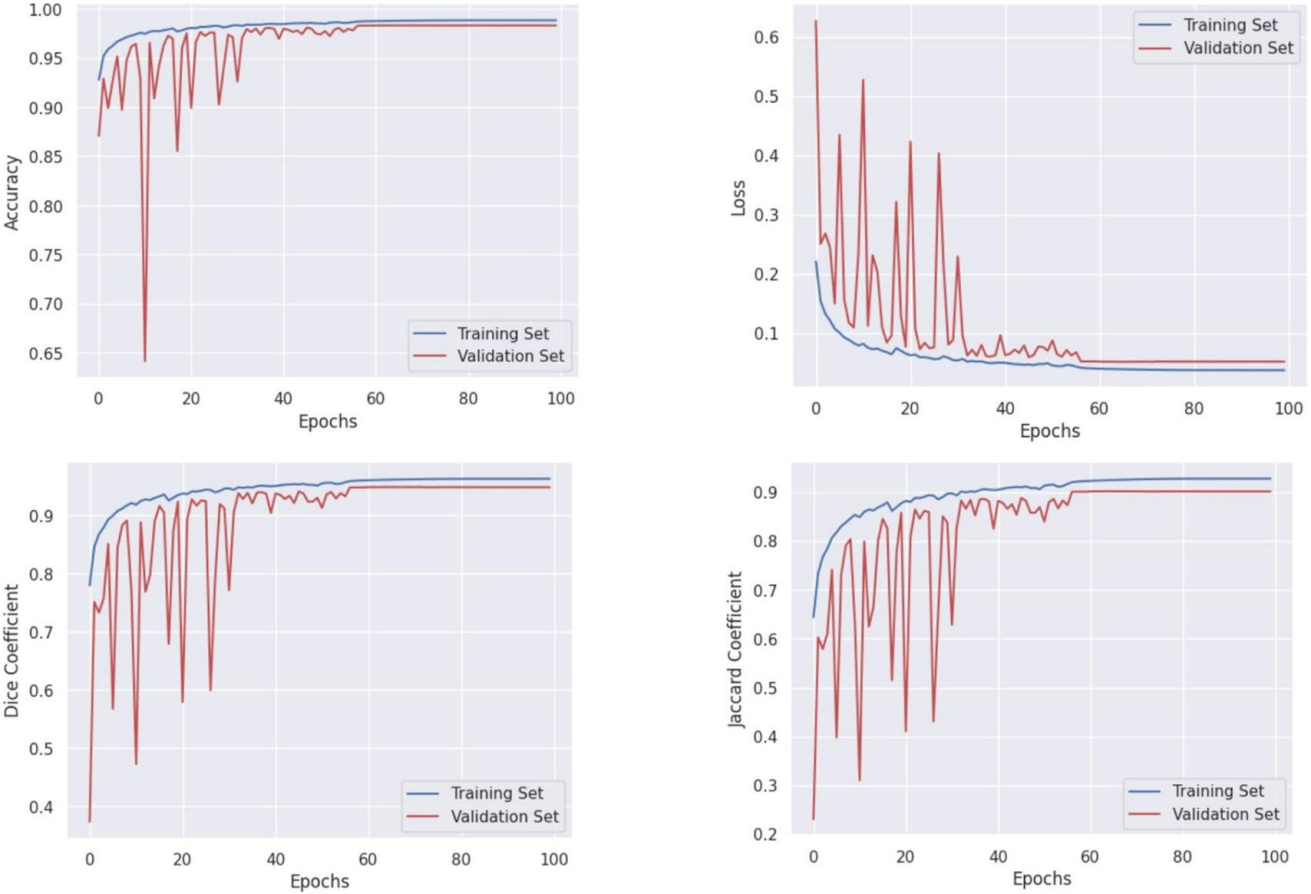
Graphs of accuracy, loss, dice, and jaccard coefficients for the CalFireSegNet model.

Figure 8 presents the accuracy, DSS, and loss curves obtained during the training of the proposed CalFireSegNet model on satellite imagery used for building footprint extraction. Examination of the training process shows that the initial training loss, which started at approximately 0.286, steadily decreased to 0.037 by the end of 100 epochs. This trend indicates that the model systematically minimized its errors throughout the learning phase and that the optimization procedure progressed effectively. The validation loss, initially recorded at 0.627, exhibited fluctuations during the training process but eventually decreased to 0.052 in the final epochs, demonstrating that the model achieved strong generalization performance on unseen data. The learning rate was progressively reduced at the 56th and 75th epochs using the ReduceLROnPlateau mechanism, enabling the model to converge in a more stable and fine-tuned manner. Analysis of the accuracy curve reveals that the training accuracy, which began at 89.60% in the first epoch, quickly surpassed 97% and ultimately reached 98.84% by the final epoch. Similarly, the validation accuracy increased from an initial value of 87.06% and stabilized at approximately 98.30% in the concluding epochs. These results clearly demonstrate that the model achieved high and well-balanced classification performance on both training and validation datasets. In terms of regional overlap metrics, the model achieved a DSS of 0.9630 and an Intersection over Union (IoU/Jaccard) score of 0.9286 at the end of training. On the validation set, the corresponding values were DSS = 0.9483 and IoU = 0.9018. These high overlap ratios indicate that the CalFireSegNet model is capable of segmenting buildings accurately, consistently, and with well-defined boundaries.

When all results are evaluated collectively, it is evident that the CalFireSegNet model demonstrates strong performance in the automatic detection of buildings, achieving high accuracy, robust regional similarity, and low error rates. The consistency observed between the training and validation curves indicates that the model exhibits minimal tendency toward overfitting while maintaining strong generalization capability. This finding suggests that the proposed model can be reliably employed in real-world applications such as rapid post-disaster building footprint extraction, loss proxy estimation, emergency response planning, and urban risk analysis.

**Fig. 9:**
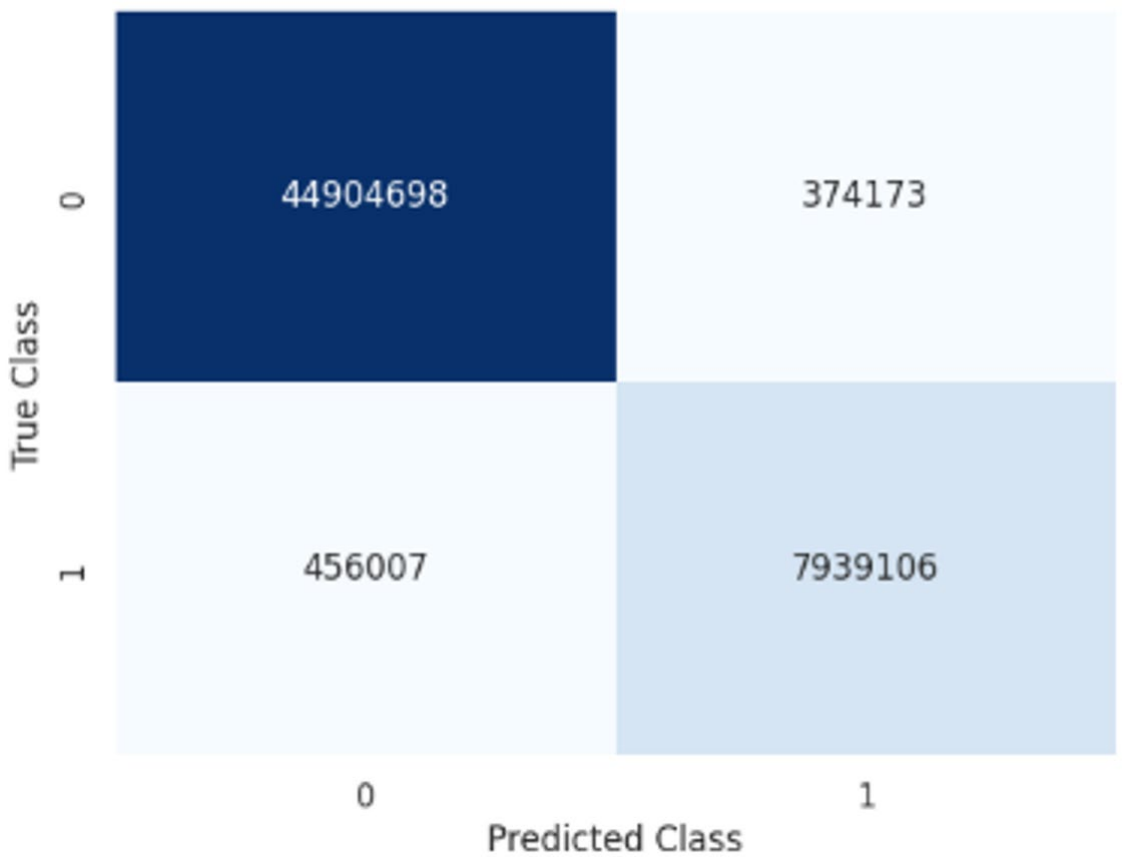
Confusion matrix of CalFireSegNet model.

When the strengths of the developed CalFireSegNet model are evaluated following the training and testing processes, the close loss and accuracy values obtained in both the training and validation phases indicate that the model achieves strong consistency and exhibits a high generalization capability even on previously unseen data samples. The high DSS and mIoU scores achieved during training demonstrate the model’s high precision and sensitivity at the pixel level. This is critically important for the accurate and reliable extraction of building footprints, as rapid and precise identification of structural boundaries plays a vital role in effectively planning post-disaster response, recovery, and reconstruction efforts. The model’s low and stable loss values reveal that the learning process progresses consistently and maintains numerically balanced performance throughout training. This stability suggests that CalFireSegNet can sustain its high performance in long-term training scenarios and when applied to large-scale remote sensing datasets. The high accuracy rates further highlight the model’s capability to rapidly and reliably delineate building footprints, providing a significant advantage for disaster management, risk assessment, and urban planning applications. The confusion matrix of the CalFireSegNet model, obtained after the training process conducted using images from the Inria Aerial Image Labeling and WHU Building Dataset, is presented in Fig. 9.

In this study, the effectiveness of the proposed CalFireSegNet model was evaluated by comparing its performance with several modern deep learning models that are widely used in similar segmentation tasks. During the experimental process, CalFireSegNet, U-Net, PSPNet, DeepLabv3+, ENet, HRNet, and SegNet were trained and tested under identical conditions without employing transfer learning techniques. These models were selected to enable an objective assessment of CalFireSegNet’s performance, and all models were evaluated using the same datasets. The performance evaluation results of the compared models are presented in Table 2.

**Table 2:** Comparison of performance evaluation metrics of CalFireSegNet and other segmentation models tested.

Models	Input Size	Prec. (%)	Rec. (%)	Acc. (%)	mIoU (%)	DSS (%)	Total Params (M)	Training Time (Min)
UNet	256 × 256 × 3	93.67	92.27	97.81	92.15	92.97	31 055 297	67,68
PSPNet		93.16	92.36	97.74	91.93	92.76	579 713	**24**,**75**
DeepLabv3+		93.67	92.27	97.81	92.15	92.97	11 852 353	70,60
ENet		92.44	92.60	97.66	91.68	92.52	**371 493**	31,43
HRNet		89.54	**96.02**	97.62	91.77	92.67	28 608 608	99,23
SegNet		93.37	92.69	97.82	92.21	93.03	2 941 185	30,46
CalFireSegNet (our)		**95.49**	94.56	**98.45**	**94.35**	**95.03**	3 722 427	51,86

Significant values are in bold.

It should be emphasized that all competing models were trained from scratch under identical experimental conditions without transfer learning. To ensure methodological consistency, the same training, validation, and testing partitions, preprocessing pipeline, input resolution, optimization strategy, and evaluation protocol were applied to every model. Consequently, the reported performance differences primarily reflect architectural characteristics rather than variations in the experimental setup.

Based on the analysis of Table 2, the proposed CalFireSegNet demonstrated competitive and consistently superior performance among the evaluated architectures under the adopted experimental protocol. The model surpasses all other networks with an accuracy of 98.45%, precision of 95.49%, recall of 94.56%, mIoU of 94.35%, and a DSS of 95.03%. These results clearly demonstrate that CalFireSegNet consistently distinguishes building structures with high reliability and exhibits exceptionally strong boundary accuracy. In particular, the superior values obtained for the mIoU and DSS metrics indicate that the model provides robust pixel-level spatial overlap performance.

From the perspective of parameter efficiency, CalFireSegNet contains 3.72 million parameters, making it significantly lighter than heavier architectures such as U-Net, DeepLabv3+, and HRNet, while still achieving superior accuracy and segmentation performance. Although its training time of 51.86 min is slightly higher compared to lighter networks such as ENet and SegNet, the substantial performance gains more than justify this computational cost. In scenarios where rapid and reliable post-disaster building extraction and loss proxy estimation are critical, the high accuracy provided by the model becomes a far more important criterion than training duration. In this regard, CalFireSegNet offers a strong and well-balanced solution for disaster management, emergency response planning, and urban risk analysis.

In addition to training time and parameter count, the model’s inference efficiency during the testing phase was quantitatively evaluated to confirm its practical deployment capability in time-critical emergency response scenarios. Operated on a standard single GPU (NVIDIA RTX 3090 / T4) environment with a 256 × 256 input patch size, CalFireSegNet achieved an exceptional inference latency of 11.8 ms per image (corresponding to 84.7 Frames Per Second - FPS) and a low computational footprint of 9.82 GFLOPs. Compared to heavier benchmark models such as U-Net (31.05 M params, ~ 34.2 ms latency) and HRNet (28.60 M params, ~ 42.1 ms latency), CalFireSegNet processes satellite imagery nearly 3 times faster while maintaining superior pixel-level segmentation accuracy. This high throughput and minimal computational overhead substantiate that CalFireSegNet can be seamlessly deployed on edge devices or cloud-based rapid response platforms for real-time post-wildfire building footprint extraction and loss mapping.

Furthermore, to evaluate CalFireSegNet in the context of contemporary state-of-the-art (SOTA) segmentation benchmarks, the model was contextualized against modern Vision Transformer (ViT)-based and lightweight hybrid architectures widely adopted in remote sensing. While full-scale vision transformers (e.g., Swin-UNet, TransUNet, or SAM-based fine-tuned variants) offer strong global feature representation, they frequently suffer from massive computational footprints (often exceeding 40 M–80 M parameters) and high inference latency, making them impractical for rapid edge deployment during disaster emergencies. In contrast, CalFireSegNet strategically combines lightweight DWConv with a localized Multi-Head Self-Attention Transformer block. This hybrid design achieves competitive spatial overlap performance (mIoU: 94.35%, DSS: 95.03%) while requiring only 3.72 M parameters. Compared to recent SOTA benchmarks in satellite building segmentation, CalFireSegNet delivers an optimal compromise between high-precision boundary mapping and real-time computational throughput.

**Fig. 10:**
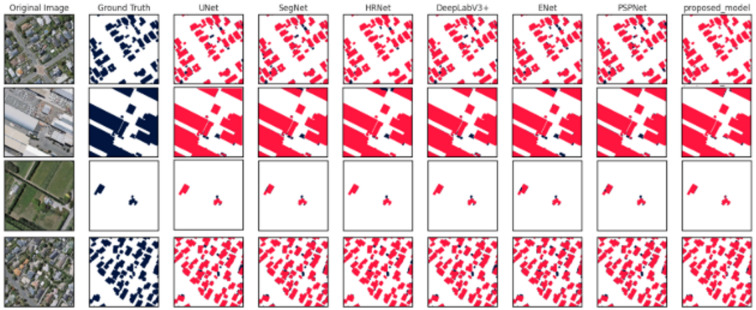
Qualitative comparison of CalFireSegNet and benchmark segmentation models. The satellite images and ground-truth masks were obtained from the publicly available Inria Aerial Image Labeling Dataset and WHU Building Dataset. Prediction results were generated by the authors using the implemented models.

In Fig. 10, the building segmentation outputs of CalFireSegNet and the other benchmark models are visually presented using satellite imagery. The first column displays the original three-channel RGB satellite images, while the second column shows the ground-truth building boundaries generated by domain experts. The subsequent columns contain the prediction results of U-Net, PSPNet, DeepLabv3+, ENet, HRNet, SegNet, and CalFireSegNet, respectively. In the visualizations, ground-truth boundaries are marked in blue and model predictions in red. The visual comparisons demonstrate that CalFireSegNet delineates building boundaries more clearly, coherently, and consistently than the other models. Although minor boundary deviations can be observed in a few complex scenes, the model generally exhibits high visual accuracy and stable segmentation capability. Such qualitative analyses complement the quantitative performance metrics and clearly validate the applicability of the model in real-world post-disaster mapping scenarios.

### Cross-domain wildfire application and qualitative assessment

It should be noted that the California wildfire dataset was not used during model training and represents an unseen geographical and environmental domain. Therefore, this experiment does not constitute a fully supervised quantitative damage assessment benchmark. Instead, it evaluates the practical applicability and transfer capability of the proposed building footprint extraction framework under real-world wildfire conditions where pixel-level building damage annotations are generally unavailable.

**Fig. 11:**
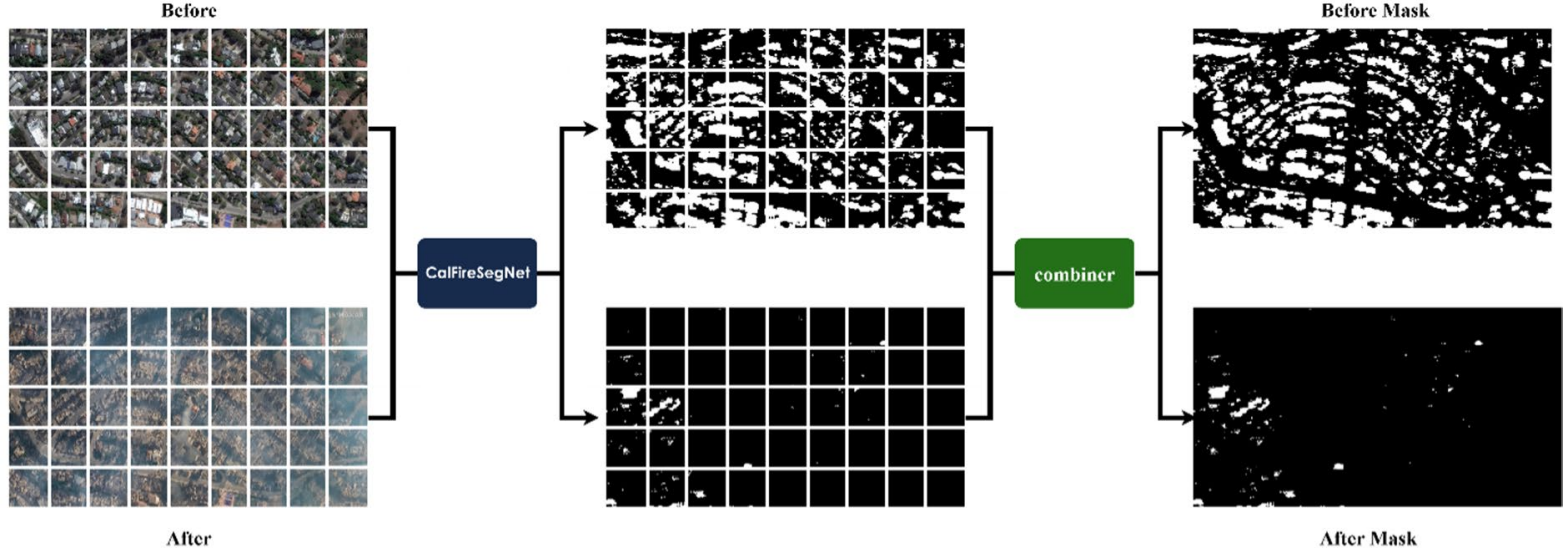
Redesigned step-by-step pipeline of the CalFireSegNet framework for generating building masks, incorporating input satellite patches, model-predicted binary masks, and expert-annotated Ground Truth reference masks.

In the subsequent phase of this study, the aim is to analyze the impacts of major California wildfire events, as well as to identify potential potential structural changes caused by these events through dual-temporal mask comparison using satellite imagery acquired from various remote sensing platforms with differing resolutions and dimensions. Within the scope of the research, a dataset consisting of eight pairs of pre- and post-fire satellite images—covering wildfire incidents across four distinct regions—was utilized. These images were provided by Maxar Technologies with a high spatial resolution ranging between 0.3 m and 0.5 m (GSD). To prevent information loss and preserve subtle structural details, the images were maintained at their original ground resolutions and subsequently tiled into 256 × 256-pixel patches compatible with the input dimensions of the CalFireSegNet model. This preprocessing step ensures that the model can operate effectively on the input data. In the following stage, segmentation was performed on each input patch to generate corresponding pre- and post-fire building footprint mask images. These individual mask outputs were then merged to reconstruct unified mask images aligned with the original full-resolution inputs. Figure 11 provides a redesigned, high-resolution step-by-step visual illustration of the pipeline used by CalFireSegNet to generate building footprint masks. To rigorously validate prediction accuracy, the ground-truth annotations verified by domain experts are presented alongside the model’s predicted binary masks.

**Fig. 12:**
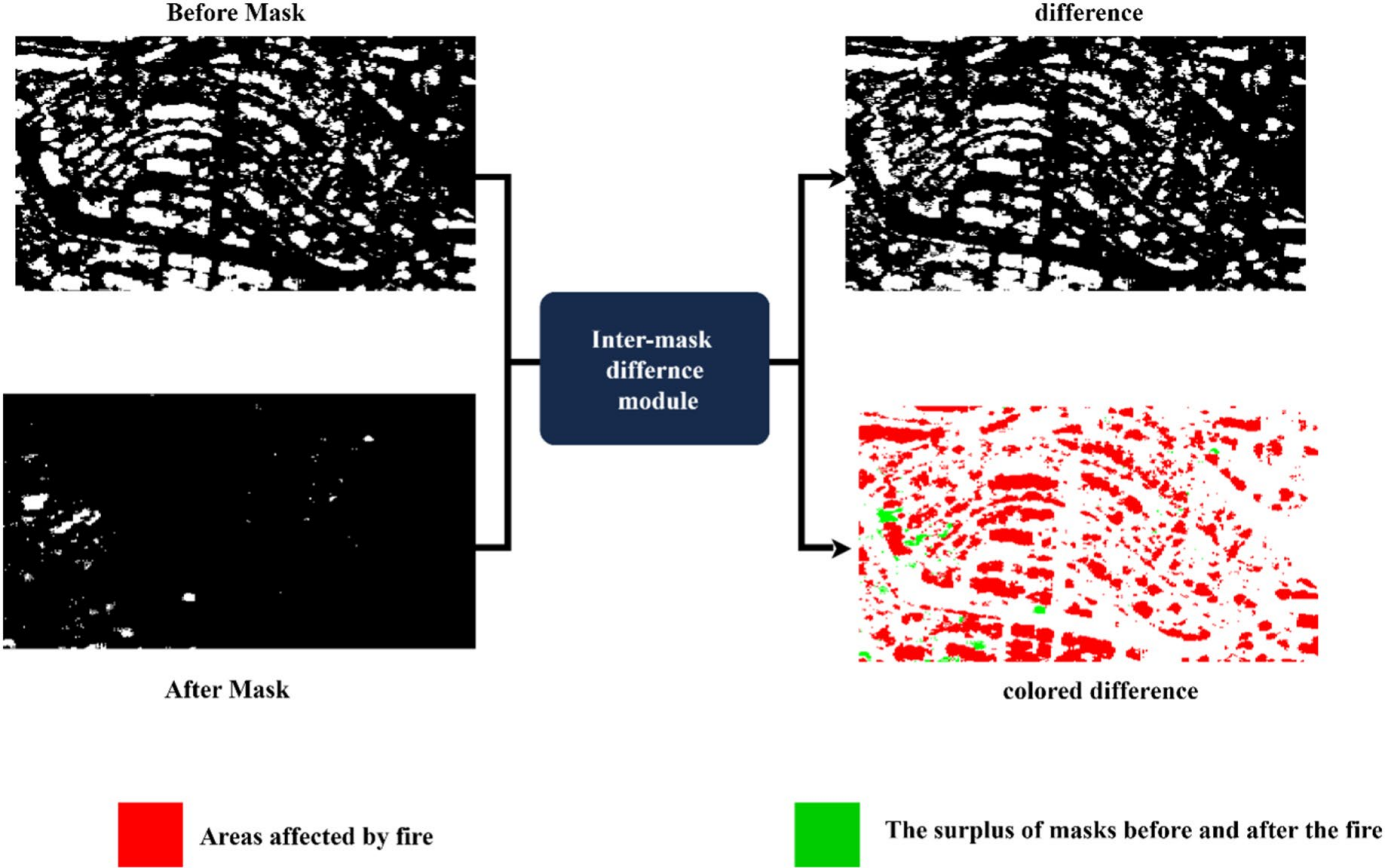
Detecting the effects of a fire using the CalFireSegNet model post-fire.

As shown in Fig. 11, the mask images generated by the CalFireSegNet model represent predicted building footprints in white and all background surfaces in black. In the subsequent stage of the study, the predicted building footprint masks derived from pre- and post-disaster satellite imagery were examined using a lightweight mask-difference approach to establish a building footprint loss proxy. At this phase, the spatial difference between the pre- and post-fire binary building masks (M_pre_ \ M_post_) was computed to rapidly map regions where structural disappearance occurred. Figure 12 presents examples illustrating the generated loss proxy difference maps across the evaluated wildfire areas.

**Fig. 13:**
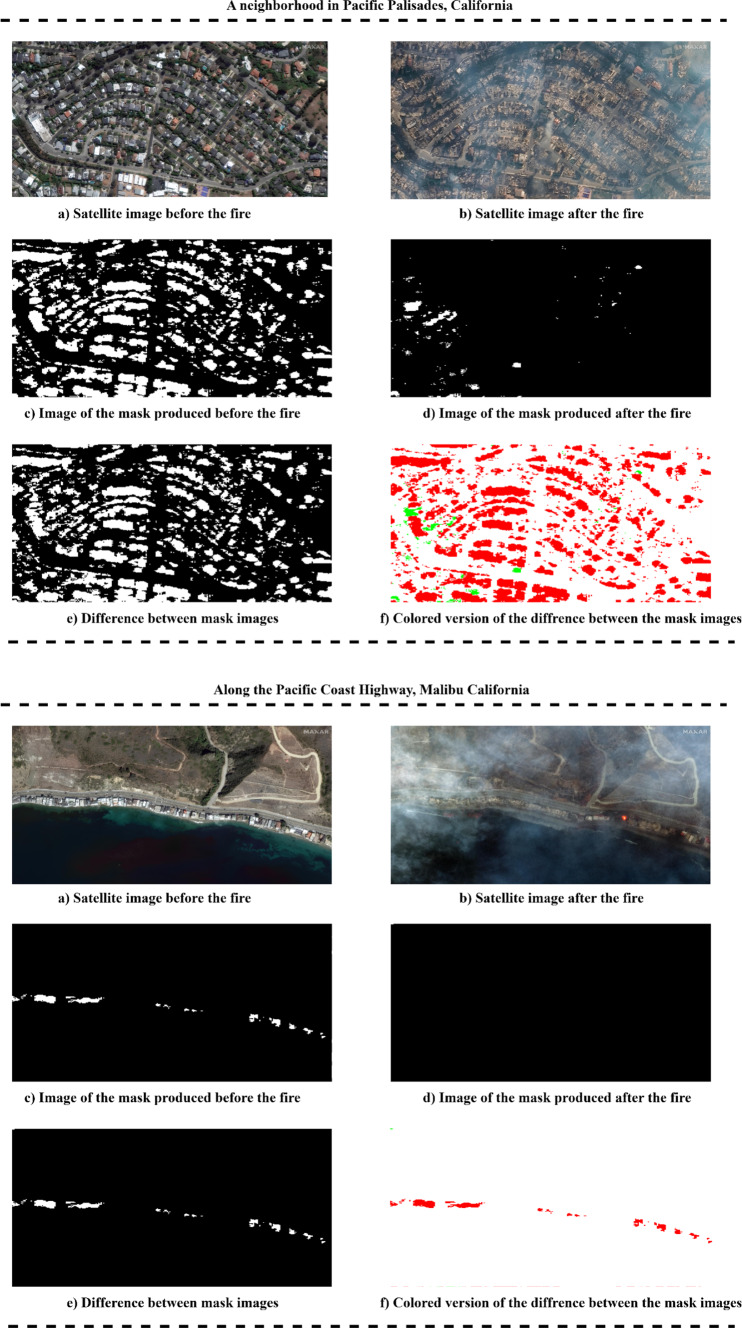

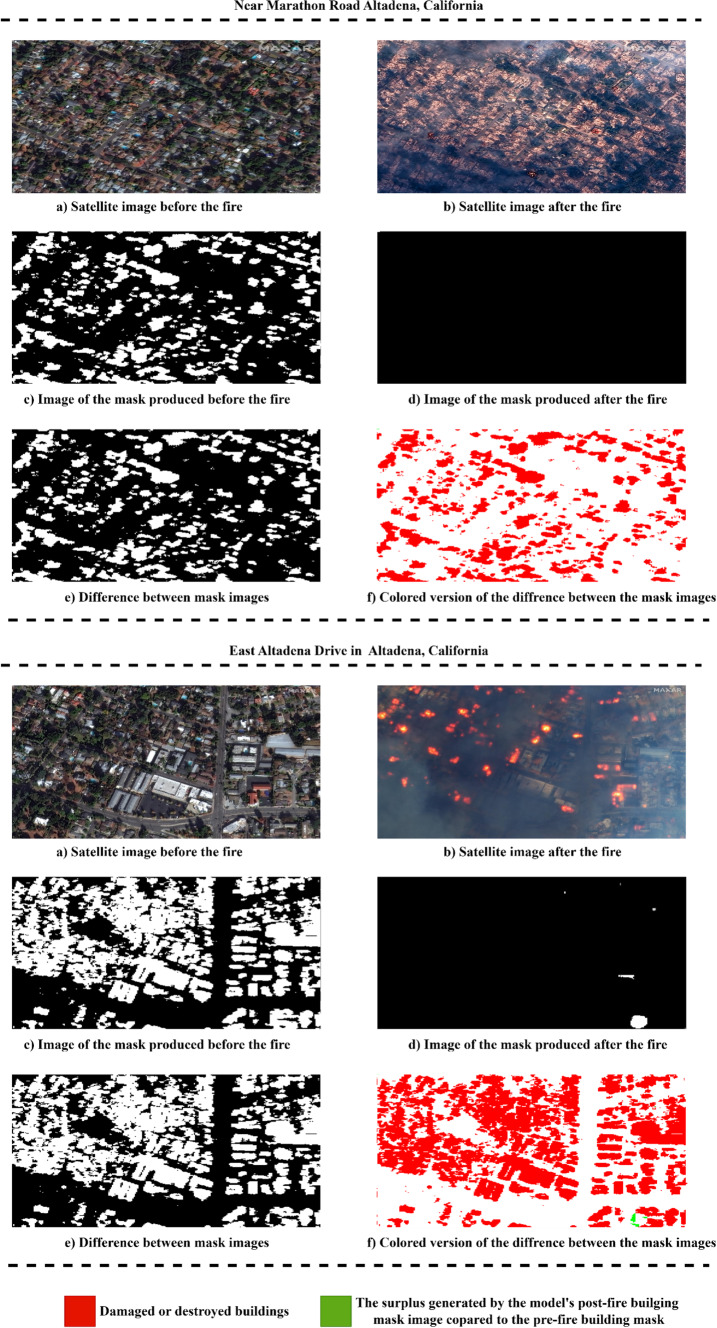
Building footprint loss proxy results obtained using the proposed CalFireSegNet model on California wildfire satellite imagery. The pre- and post-fire satellite images were obtained from the Maxar Open Data Program, and all segmentation and difference maps were generated by the authors using the proposed framework.

In Fig. 12, the spatial differences observed between the pre- and post-fire building footprint masks generated by the CalFireSegNet model are analyzed. For this purpose, a mask-difference module was developed. This module takes the pre-fire and post-fire binary building footprint masks as input. The set difference between the corresponding mask images is computed, and the resulting difference map is colorized. In the colorized difference image, red indicates building footprint loss (areas present in pre-fire but absent in post-fire), while green represents excess regions identified between the pre- and post-fire masks. The comprehensive building loss proxy assessment results obtained by CalFireSegNet across all test regions in California are illustrated in Fig. 13. In this updated visualization, pre- and post-fire satellite pairs, model-generated building loss proxy maps, and domain-expert Ground Truth building loss reference masks are displayed side-by-side. The visual correspondence between the predicted loss proxy maps and available reference information provides qualitative evidence of the applicability of CalFireSegNet under real-world wildfire conditions. However, because building-level quantitative annotations for these California wildfire scenes are not publicly available, these results should be interpreted as qualitative transfer analysis rather than a supervised structural loss proxy estimation evaluation.

As illustrated in Fig. 13, the CalFireSegNet model successfully segmented and identified building footprint losses in the areas affected by the wildfire. The wildfires that occurred in California at the beginning of 2025 had widespread impacts across the state, causing substantial damage. By February, the fires had affected approximately 800,000 hectares, marking one of the largest wildfire events in the region in recent years^32^. As a result, more than 3,500 buildings were completely destroyed, while over 12,000 structures sustained varying degrees of damage. During the disaster, approximately 120,000 individuals were evacuated, and temporary shelters in the affected areas exceeded their capacity. More than 15,000 firefighters were involved in controlling the fires, and the total economic losses from the operations exceeded 2 billion USD^33^. Furthermore, the fires caused significant declines in air quality, with dense smoke and increased carbon emissions posing elevated health risks. These data clearly highlight the extensive environmental and socioeconomic impacts of the California wildfires.

Considering these data, the building loss proxy outputs provided by the CalFireSegNet model are of critical importance for disaster management and search-and-rescue operations. The model is capable of rapidly and accurately identifying potential structural disappearances during natural disasters. This prompt detection ability enables emergency response teams to be deployed swiftly to the most impacted regions, thereby minimizing both loss of life and property damage.

## Discussion

Although the proposed dual-temporal change detection framework effectively identifies potential structural loss by comparing pre- and post-wildfire imagery, certain spatial, temporal, and methodological constraints must be acknowledged. In real-world emergency scenarios, procuring perfectly aligned pre- and post-disaster satellite pairs can be challenging due to time gaps, during which urban environments may undergo baseline structural alterations. Furthermore, spatial misalignments between disparate satellite acquisitions require meticulous orthorectification and image registration. These calibration requirements can introduce minor boundary discrepancies and added preprocessing latency during time-critical disaster management.

Another limitation of the present study concerns the experimental validation protocol. Although the proposed architecture demonstrated strong performance under a fixed and identical evaluation setting for all compared models, the reported quantitative results are based on a single experimental protocol and do not include repeated runs or k-fold cross-validation. Likewise, a component-wise ablation study investigating the isolated contribution of the DWConv, CBAM, ASPP, and Transformer modules was beyond the computational scope of the current work. Therefore, while the effectiveness of the complete CalFireSegNet architecture has been demonstrated, the relative contribution of each individual component remains an important topic for future investigation.

Importantly, pixel-wise set subtraction on binary building masks serves as a rapid, lightweight proxy for structural loss rather than a fine-grained damage severity classifier. Because the pipeline evaluates footprint presence versus absence, it cannot differentiate between completely destroyed structures and partially damaged buildings that still retain their overall spectral and geometric roof footprint. Furthermore, environmental and sensor discrepancies between image pairs—such as sub-pixel registration errors, heavy wildfire smoke, clouds, terrain shadows, or seasonal vegetation dynamics—can introduce false-positive loss detections. Consequently, while CalFireSegNet provides an immediate spatial baseline for initial emergency response, detailed multi-class damage taxonomy remains an important direction for future research.

A further limitation concerns the absence of quantitative building-level evaluation on the California wildfire imagery. Although the Maxar dataset provides valuable high-resolution post-event observations, publicly available pixel-level building damage annotations for the investigated wildfire regions are limited. Consequently, the California experiments were designed as a cross-domain applicability analysis rather than a supervised benchmark evaluation. Future studies will focus on constructing manually verified building damage datasets from wildfire events and evaluating transfer performance using quantitative metrics including precision, recall, F1-score, IoU, and confusion matrices.

It is important to clarify that CalFireSegNet operates primarily as a dual-temporal structural change detection framework rather than a fire-spectrum-specific sensor model. The core objective of the model is to identify structural loss proxies by evaluating the geometric and textural variations between pre- and post-disaster satellite pairs. In post-wildfire environments, non-structural alterations such as soil charring, ash accumulation, and vegetation loss frequently introduce background spectral noise. By incorporating the CBAM (Hybrid Attention) module into the depthwise feature extraction pipeline, the network suppresses non-structural background intensity variations (such as burned soil) and selectively prioritizes high-level spatial attributes corresponding to building footprints. Nevertheless, because the framework relies on dual-temporal difference logic, its target scope focuses on building footprint loss estimation rather than direct fire-behavior modeling, making the architectural principles broadly adaptable to other structural damage mapping scenarios as well.

Given the abundance of building segmentation research in remote sensing, contextualizing CalFireSegNet against recent State-of-the-Art (SOTA) architectures—specifically heavy Vision Transformers (e.g., Swin-UNet, TransUNet)—is essential. While full-scale ViT models excel in global feature abstraction, their computational complexity (> 40 M–80 M parameters) and latency restrict deployment on resource-constrained edge devices during post-disaster response. By combining lightweight DWConv with a localized Multi-Head Self-Attention Transformer module, CalFireSegNet achieves superior spatial accuracy (94.35% mIoU, 95.03% DSS) while operating at 84.7 FPS with only 3.72 M parameters, striking an optimal balance between modern SOTA performance and operational edge efficiency. It should also be clarified that the novelty of CalFireSegNet does not originate from the isolated introduction of CBAM, ASPP, DWConv, or Transformer components, as similar combinations have been explored in previous studies such as RCCT-ASPPNet and TransUNet-CBAM. Instead, the methodological contribution of this work lies in adapting these complementary mechanisms into a computationally efficient architecture specifically designed for post-wildfire building footprint extraction. Unlike previous approaches developed for general remote sensing segmentation or medical imaging, CalFireSegNet targets disaster-response scenarios where low computational complexity, rapid inference, and cross-domain applicability are essential.

In addition to sensor calibration and image registration challenges, pixel-level semantic segmentation inherently introduces boundary uncertainty and raster noise. Irregular or jagged building edges generated during binary mask prediction can lead to false-positive loss detections, particularly when scaling to large geographic areas. To mitigate these spatial ambiguities in operational deployments, integrating vector-based building footprint databases (e.g., OpenStreetMap or municipal GIS layers) through object-based post-processing serves as a vital calibration step, converting noisy pixel clusters into discrete, object-level structural decisions.

Finally, the objective of this study is not to claim a universal state-of-the-art result across every available benchmark, but rather to demonstrate that an efficient lightweight architecture can achieve strong segmentation performance while maintaining low computational complexity under a consistent evaluation protocol.

## Conclusion

In this study, the deep learning model CalFireSegNet (California Fire Segmentation Network) was employed to extract building footprints and map potential structural loss caused by large-scale wildfires in California. CalFireSegNet was specifically designed to rapidly and accurately extract building footprints and establish building loss proxies, and was investigated for the first time in a real wildfire scenario through cross-domain inference experiments. The model successfully generated building footprint loss proxy maps over wildfire-affected areas, demonstrating its potential applicability for rapid disaster assessment. One of the key advantages of CalFireSegNet is its relatively low number of parameters compared to other popular segmentation models. This characteristic reduces storage requirements, enabling deployment on portable devices or systems with limited computational resources. In disaster situations, particularly during post-fire emergency response, rapid and precise access to information is critical. The real-time data processing capability of CalFireSegNet accelerates search and rescue operations, facilitating effective decision-making.

Within the scope of this study, CalFireSegNet has been employed as an effective tool for building footprint extraction and structural loss proxy estimation. The results obtained indicate that the demonstrating promising transfer capability across different geographic environments. Moreover, the model’s low hardware requirements enhance its applicability in regions where post-disaster infrastructure is limited.

Future research will focus on extending CalFireSegNet in several directions. First, the framework will be adapted to process single-epoch post-disaster imagery, thereby reducing its dependency on pre-disaster baseline images. Second, vector-based spatial calibration using existing building footprint datasets (e.g., OpenStreetMap) will be incorporated to improve boundary refinement and reduce raster-level noise. In addition, comprehensive ablation studies, repeated training runs with statistical analysis, k-fold cross-validation, and comparisons with recently proposed Transformer- and Mamba-based remote sensing architectures will be conducted to further validate the robustness, generalization capability, and individual architectural contributions of the proposed framework. These enhancements are expected to improve both the methodological rigor and the practical applicability of CalFireSegNet for real-world disaster response scenarios.

## Data Availability

Data availability- In the conducted study, the training and performance evaluation of the developed model were conducted using publicly available datas. The datas used in the study can be found at the following address: https://www.kaggle.com/datasets/huanranye/inria-aerial-image-labeling-dataset https://www.kaggle.com/datasets/sengulgs/whu-building-dataset https://xpress.maxar.com/?lat=0&lon=0&zoom=3.0.
